# Cellular and humoral peritoneal immunity to *Mesocestoides vogae* metacestode infection in mice

**DOI:** 10.1186/s13071-020-04541-0

**Published:** 2021-01-18

**Authors:** Terézia Mačak Kubašková, Dagmar Mudroňová, Miroslava Vargová, Katarína Reiterová, Gabriela Hrčková

**Affiliations:** 1 Institute of Parasitology of the Slovak Academy of Sciences, Hlinkova 3, 040 01 Košice, Slovakia; 2 The University of Veterinary Medicine and Pharmacy in Košice, Komenského 68/73, 040 01 Košice, Slovakia

**Keywords:** Experimental larval cestodiasis, *Mesocestoides vogae*, Host’s immune response, Peritoneal cavity

## Abstract

**Background:**

Here, *Mesocestoides (M.) vogae* infection in mice is proposed as a suitable experimental model for studying the immunity in the peritoneal cavity of mice.

**Methods:**

To investigate the kinetics of immune parameters in *M. vogae*-infected mice, we detected, using flow cytometry, the expression of selected lymphoid and myeloid markers within the peritoneal cell population at day 0, 3, 6, 10, 14, 19, 25, 30 and 35 post-infection. Then, using ELISA, we analyzed the cytokine IFN-γ, TGF-β, IL-4 and IL-10 responses and the levels of anti-*M. vogae* IgG and IgM antibodies in the peritoneal lavage fluid. Cells isolated from the peritoneal cavity were subjected to further molecular analysis. To assess cell activation, peritoneal cells were exposed to LPS, and culture supernatants were collected and assayed for the level of cytokines and production of nitrite. Ly6C+ and Ly6G+ cells were isolated using MACS from the peritoneal cells at day 35 post-infection. Both MACS-isolated subsets were co-cultured with preactivated T cells to measure their suppressive capacity. Next, the role of parasite excretory-secretory antigens in induction of CD11b+ myeloid cells with the suppressive phenotype and the production of IL-10 was examined.

**Results:**

In the peritoneal cavity an initial increase of CD11b+Gr-1+F4/80^high^MHC II^high^ cells, NK, NKT cells and CD8+ cytotoxic T cells was observed in the first week of infection. At day 14 post-infection, an increase in the number of myeloid CD11b+Gr-1+ cells was detected, and most of this cell population expressed low levels of F4/80 and MHC II in later stages of infection, suggesting the impairment of antigen-presenting cell functions, probably through the excretory-secretory molecules. Moreover, we confirmed that peritoneal Gr1+ cells (Ly6C+ and Ly6G+ population) are phenotypically and functionally consistent with myeloid-derived suppressor cells. Metacestode infection elicited high levels of IL-10 and upregulated STAT-3 in peritoneal cells. A higher level of IgM suggests that this isotype may be predominant and is involved in the host protection.

**Conclusions:**

*Mesocestoides vogae* tetrathyridia induced the recruitment of immunosuppressive cell subsets, which may play a key role in the downregulation of immune response in long-term parasitic diseases, and excretory-secretory antigens seem to be the main regulatory factor.
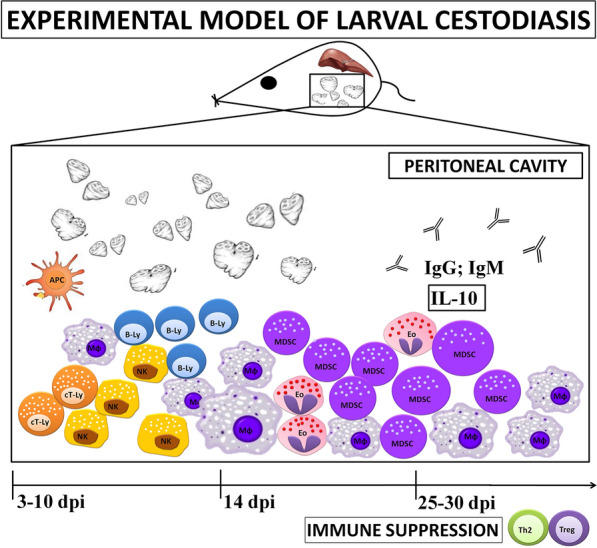

## Background

Larval cestodiases are zoonotic diseases caused by the larval stages of tapeworms. They represent infections of veterinary, medical and economic importance with a worldwide distribution. Metacestodes of tapeworms belonging to the family Taeniidae may develop in various tissues or serosal cavities of intermediate hosts, causing serious damage with a fibrous change of the surrounding tissue. A prominent characteristic of the host-parasite interplay in these infections is the dampening of the host’s immune mechanisms actively driven by their excretory-secretory (ES) molecules [1].

*Mesocestoides* spp. adults, a parasitic agent of the gastrointestinal tract of carnivores, can in rare cases cause intestinal infection in humans. Like most cestode species, their life cycle represents a predator-prey relationship. Metacestodes of the tapeworm *Mesocestoides (M.) vogae* (second larval stage) (syn. *M. corti*) can multiply asexually in the liver and peritoneal cavity of their intermediate hosts, which include amphibians, reptiles and rodents. Oral infection of mice represents a research model for investigating the various aspects of cestode biology or pathology [2]. Invasion of the hepatic tissue by metacestodes results in extensive parenchymal destruction with fibrosis and the development of ascites. To date, the experimental model of larval cestodiasis has been preferentially used to study host-parasite interactions, specifically in the liver [3] or the peritoneum [4]. Intracranial inoculation of metacestodes serves as a laboratory model of neurocysticercosis to study immunity and metacestodes-associated pathology in the brain [5, 6]. Infection with *M. vogae* tetrathyridia as a model parasite offers considerable potential for experimental immunological and pharmacological studies of medically important metacestodes of *Echinococcus* spp. or *Taenia* spp.

The modulation of immune response is a key component of *M. vogae* pathology. Only a few previous studies have investigated immune-related host events, but the mechanisms that provide long-lasting protection for parasites are still unclear. It has been reported that intraperitoneal administration of IFN-γ reduces the parasite burden in mice [7], suggesting a critical role of Th1 immunity. However, increased Th1 cytokines during *M. corti* (*M. vogae*) infection is associated with lethality of IL-4^−^/^−^ mice and impairment of alternatively activated macrophages [8]. Recently, a central role of larval ES products in host immunomodulation by *M. vogae* was demonstrated [4, 9].

The microenvironment in the peritoneal cavity can be manipulated by metacestodes by recruiting immune cells and regulating the cytokine balance. The multiplication of metacestodes in experimental larval cestodiasis is a peritoneal phenomenon; therefore, we examined the cellular populations and various immune events in the peritoneal cavity of infected mice at different points in time. We observed that *M. vogae* infection induced a marked expansion of CD11b+Gr1+ cells phenotypically and functionally consistent with myeloid-derived suppressor cells (MDSC). In the first 2 weeks of infection, the number of cytotoxic CD8 T cells and NK cells increased, and peritoneal exudate cells were able to respond to LPS by the production of inflammatory mediators, suggesting the presence of classically activated cells. At later points in time, IL-10 cytokine remained predominant in the peritoneum with apparent abrogation of the IFNγ/IL-4 signaling pathway. Moreover, ES antigens released by *M. vogae* tetrathyridia are essential for recruitment of myeloid cells and immunoregulatory mediators.

## Methods

### Animal infection and experiment design

*Mesocestoides vogae* infection is maintained by serial passage from infected mice to a naive ICR-strain of mice at the animal facilities of the Institute of Parasitology of the Slovak Academy of Sciences under pathogen-free conditions. Metacestodes of *M. vogae* were collected in sterile physiological saline from the peritoneal cavity of female mice after 2–4 months of infection.

The experiment was carried out on 8-week-old male BALB/c mice purchased from Velaz (Prague, Czech Republic). The mice were infected orally (60 ± 5 tetrathyridia per animal) and randomly divided into eight groups (*n* = 5 per group). Healthy mice were used as a control (*n* = 3). Peritoneal exudates as well as peritoneal exudate cells (PEC) were obtained on day 0, 3, 6, 10, 14, 19, 25, 30 and 35 post-infection (p.i.). In the subsequent experiments aimed at studying the direct effect of parasitic ES antigens, groups of mice (*n* = 5/each) received either intraperitoneal injections of 200 µl of PBS (control) or 20 µg of ES in 200 µl of PBS and the applications of ES and PBS were performed six times, every day for a total 6 days. The antigen doses correlated approximately with the concentration of *M. vogae* secretion detected in vivo [4]. In these experiments, peritoneal fluid and PEC were isolated as described below and used in further analysis.

### Isolation of exudates and cell preparation

Exudates from peritoneal cavities of healthy and infected mice were collected by washing the peritoneal cavity with 1 ml of sterile PBS, and PEC were obtained by second peritoneal lavage under sterile conditions by injections of 5 ml of Roswell Park Memorial Institute-1640 (RPMI) medium (Biochrom, Berlin, Germany) containing 2 mM of stable glutamine and supplemented with 10% heat-inactivated bovine fetal serum (Biochrom, Berlin, Germany), 100 U/ml penicillin, 100 μg/ml streptomycin, 10 μg/ml gentamicin and 2.5 μg/ml amphotericin B (complete medium, CM) (all from Sigma-Aldrich, St. Louis, MO, USA). The freshly isolated cell suspension was then washed with LPS-free Dulbecco phosphate-buffered saline (DPBS, Sigma-Aldrich, St. Louis, MO, USA) and resuspended in CM. The viability and numbers of isolated cells were evaluated by trypan blue exclusion test (Sigma-Aldrich, St. Louis, MO, USA). Then, PECs were stained with various combinations of antibodies, and the cell suspension was subsequently aliquoted for phenotypic analysis by flow cytometry and an assessment of classical cell activation (LPS stimulation of PEC). The remainder of the unstained cell samples was utilized for RNA extraction.

To evaluate the suppressive capacity of the peritoneal myeloid cell subsets, Ly6G+ and Ly6C+ cells were separated from the PEC of infected mice at day 35 p.i. Ly6G+ cells were isolated by positive selection using biotin conjugated anti-mouse Ly6G antibody and magnetic streptavidin nanobeads (MojoSort Mouse Ly-6G Selection Kit, Biolegend, San Diego, CA, USA). The magnetically labeled fraction was separated on a MACS column according to the manufacturer’s instructions. Ly6C+ cells were collected from Ly6G-depleted fractions using Gr-1-biotin antibody and magnetic streptavidin nanobeads (both from Biolegend, San Diego, CA, USA). The purity of various populations was determined via FACS analysis, and the viability and numbers of isolated cells were evaluated using trypan blue staining (Sigma-Aldrich, St. Louis, MO, USA).

### May-Grünwald/Giemsa staining

To evaluate the morphology of cells, 1 × 10^5^ sorted Ly6G+ and Ly6C+ cells were fixed on glass slides using the cytospin technique. Then, the cells were dried and stained with May-Grünwald/Giemsa solutions (Sigma-Aldrich, St. Louis, MO, USA) according to the standard procedure. The stained cells were then observed under a light microscope (Olympus, Prague, Czech Republic), and an analysis of the cell types was done at 1000× magnification.

### Flow cytometric analysis

PEC were enumerated (*n* = 5/infected group; *n* = 3/control group) and resuspended in CM (0.5 × 10^6^ cells/100 μl). The viability of these cells was more than 95% determined by trypan blue exclusion. Fifty-microliter aliquots of cell suspension were plated into a round bottom tube and stained with monoclonal antibodies that recognize CD11b (FITC; clone M1/70; Biolegend, San Diego, CA, USA), F4/80 (APC; clone CI:A3-1; BioRad, Hercules, CA, USA), Gr1 (PE; clone RB6-8C5), MHC II (Pe Cyanine7; clone M5/114.15.2), CD3 (PerCPeFluor710; clone 17A2), CD4 (FITC; clone GK 1.5), CD8 (PE; clone 53-6.7) and CD 49b (APC; clone DX5) from eBioscience (San Diego, CA, USA). Cells were stained for 20 min at room temperature, fixed with fixation buffer (eBioscience, San Diego, CA, USA) for 15 min and rinsed twice with FACS buffer.

In the experiment focused on the role of the application of ES antigens in vivo on the myeloid phenotype, intracellular staining of IL-10 was performed. Briefly, isolated PEC were first stained for 30 min at 4 °C with antibodies to CD11b surface marker. After two washes with PBS, intracellular staining was performed using the Cytofix/Cytoperm kit (BD Biosciences, San Jose, CA, USA) and anti-IL-10 (PE; clone: JES5-16E3, Sony Biotechology, San Jose, CA, USA) according to the manufacturer’s instructions. Phenotypic analysis was performed on a FACS Canto (Becton Dickinson Biosciences, USA), and the acquired data were analyzed using the FACS Diva software. The portion of unstained cells was used for RNA extraction.

### T-cell suppression assay

Spleen cell preparations were prepared from naïve mice. Spleens were gently homogenized between two glasses, and each sample was spun at 1500 rpm for 5 min. The supernatant was removed, and the cells were resuspended in 5 ml of cold NH_4_Cl. Following the lysis of red blood cells, splenocytes were washed and diluted at 2 × 10^6^/ml in 5 ml of RPMI supplemented with 5% fetal bovine serum (FBS). CFSE (Biolegend, San Diego, CA, USA) was added to reach a final concentration of 2.5 µM and incubated at room temperature for 7 min. Next, Dulbecco PBS (DPBS) supplemented with 20% FBS was added. The cells were washed three times in RPMI with 10% FBS and plated. The CFSE-labeled splenocytes (at a concentration of 1 × 10^6^/ml and a volume of 100 µl) were plated in 96-well plates coated with 10 µg/ml of anti-CD3 and 1 µg/ml of anti-CD28 mAbs (Invitrogen, Carlsbad, CA, USA). The splenocytes were cultured for 3 h on Ab-coated plates, and then 100 µl of PEC (Ly6C+ or Ly6G+ cells) was added to obtain the following ratios of PEC to splenocytes: 1:1, 1:2, 1:4, 1:8, 1:16 and 1:32. Suppression assay was performed in triplicate. The co-cultures were incubated for 72 h and then harvested and analyzed by flow cytometry. Dilution of the CFSE was evaluated as a measure of T-cell proliferation by flow cytometry. To that end, cells were additionally stained with anti-CD3-PerCP-Cy5.5 (Biolegend, San Diego, CA, USA). The percentage suppression of proliferation was calculated as (1 − (proliferation with MDSC: proliferation without MDSC)) × 100.

### Cytokine detection

The concentrations of IFN-γ, IL-4, TGF-β and IL-10 present in peritoneal fluid and culture supernatants of LPS-stimulated PEC (*n* = 5/infected group; *n* = 3/control group, examined in duplicates) were quantified by commercial ELISA Kits (Mouse Ready-SET-Go ELISA, all from eBioscience, San Diego, CA, USA) according to the manufacturer’s instructions. Concentrations of cytokines were calculated in pg/ml.

### Determination of nitrite production ex vivo and cytokine level in culture supernatants

To measure the production of nitric oxide (NO) and level of cytokines by total PEC (*n* = 5/infected group; *n* = 3/control group, examined in duplicates), cell suspensions (1 × 10^6^ cells/ml) were cultured in 24-well plates (Corning) in CM in the presence or absence of LPS (1 µg/ml). The plates were incubated for 72 h at 37 °C, 5% CO_2_. The concentration of NO in the culture supernatants was determined as nitrite (NO_2_^−^) using Griess reagent. Briefly, 50 µl of a solution containing 1% sulfanilamide/5% H_3_PO_4_ was incubated with 50 µl supernatants in a 96-well plate (in triplicate) for 10 min at room temperature. After incubation, a second solution (0.1% *N*-(1-naphthyl) ethylenediamine dihydrochloride) was added to the mixture, and the absorbance was measured at 550 nm using an ELISA reader. Nitrite concentration was determined from the calibration curve using 0.1 M NaNO_3_ as standard. For determination of the levels of TNF-α, IL-6 and IL-10 by ELISA kits (Mouse Ready-SET-Go ELISA, all from eBioscience, San Diego, CA, USA), the rest of culture supernatants was collected and stored at − 20 °C until use. The supernatants were diluted 1:2 with PBS before use.

### RNA isolation and real-time PCR

Total RNA was extracted from the PEC (*n* = 5/infected group; *n* = 3/control group) using TRIzol reagent (Invitrogen, Carlsbad, CA, USA). The RNA was quantified using a NanoSpectrophotometer AstraGene (Harston, Cambridge, UK), and 3 µg was transcribed to cDNA using ReverseAid H Minus M-MuLV Reverse Transcriptase and oligodT primers (Thermo Scientific, Burlington, ON, Canada). The cDNA for each cell sample was used as a template for real-time PCR reactions. Quantitative PCR analysis of the relative abundance of mRNA species was determined using the SYBR green master mix (BioRad, Hercules, CA, USA) on a BioRad CFX thermocycler (BioRad, Hercules, CA, USA). PCR was performed in 20-μl reactions with detection primer pairs for STAT-1 (forward: 5′-CTGAATATTTCCCTCCTGGG-3′; reverse: 5′-TCCCGTACAGATGTCCATGAT-3′), STAT-3 (forward: 5′-GAAGCCGACCCAGGTGC-3′; reverse: 5′-GTCACGTCTCTGCAGCTTCT-3′), STAT-6 (forward: 5′-GAGTTCCTGGTCGGTTCAGA-3′; reverse: 5′-GCTCTCCAAGGTGCTGATGT-3′), iNOS (forward: 5′-GCCTCATGCCATTGAATTCATCAACC-3′; reverse: 5′-GAGCTGTGAATTCCAGAGCCTGAA-3′) and Arg-1 (forward:5′-CAGAAGAATGGAAGAGTCAG -3′; reverse: 5′-CAGATATGCAGGGAGTCACC-3′). Data were normalized to a housekeeping gene (β-actin), and relative quantification was done using the 2^−∆∆*C*t^ method.

### SDS‐PAGE and Western blotting

ES products or somatic homogenate (MvH) was mixed with 5× reducing sample buffer, boiled for 5 min at 95 °C and processed for SDS-PAGE. The final protein concentration in each prepared sample was 80–100 μg/100 μl, which was subsequently resolved in 12% acrylamide gels under denaturing conditions and electrophoretically transferred to nitrocellulose membranes (0.45 μm pore size, Merck Millipore Ltd., Tullagreen, Carrigtwohill, Co., Cork, Ireland) using a Mini Trans-Blot Electrophoretic Transfer Cell (Bio-Rad, Hercules, CA, USA). Strips with blotted antigens were utilized for immunodetection of specific anti-tetrathyridial IgG and IgM antibodies. Nonspecific binding was blocked upon incubation in 5% fat-free milk in PBS for 1 h, and strips were incubated with the sera diluted in 3% fat-free milk (1:50) overnight at 4 °C. The blots were washed and probed with the HRP-conjugated goat polyclonal anti-mouse IgG (ICN, USA) diluted to 1:500 for 1 h at 37 °C. Finally, the immunoreactive bands were visualized by treating the membranes with the substrate solution (4-chloronaphthol and H_2_O_2_ in PBS).

### Immunofluorescence

The suspension of peritoneal cells was placed on glass slides and fixated with 4% formaldehyde for 15 min at room temperature. Nonspecific binding was blocked by incubation with 4% goat serum for 30 min at room temperature. The cells were incubated with primary antibody against iNOS or Arg-1 (1:250) (Abcam, Cambridge, UK) for 2 h at room temperature and then washed three times with PBS. After incubation with secondary antibodies (1:500, FITC-conjugated anti-rabbit IgG-F0112, PE-conjugated anti-rabbit IgG-F0110, both from R&D Systems, Minneapolis, MN, USA) in the dark for 1 h at room temperature, nucleus staining was performed using Hoechst 33258 Staining Dye Solution (Abcam, Cambridge, UK). Images were obtained with a fluorescence microscope (Leica DM IRB, Germany).

### Determination of Ag-specific antibodies by ELISA

Parasite products (ES products or somatic homogenate MvH) were prepared as described by Vendelova et al. [4, 9], and the protein content was assessed using Bradford protein assay reagent (BioRad, Hercules, CA, USA) and using BSA (Sigma-Aldrich, St. Louis, USA) as the standard.

To determine the specific IgG/IgM, flat-bottom 96-well plates (Nunc Maxisorp) were coated with worm antigens (2.5 μg/ml ES or MvH) in carbonate-bicarbonate coating buffer (pH 9.6) per night at 4 °C. The microplates were blocked for 1 h at room temperature with 10% bovine fetal serum-PBS solution. Sera/peritoneal exudates diluted 1:100 were incubated for 1 h at 37 °C. The microplates were then washed, and goat anti-mouse IgG/IgM peroxidase conjugates diluted 1:5000/1:2000 (Sigma-Aldrich, St. Louis, USA) were added for 1 h at 37 °C. After further washes, *O*-phenylendiamine (Sigma-Aldrich, St. Louis, MO, USA) was added as a substrate, and absorbance values were recorded at 492 nm. Results were expressed as the mean optical density (OD) ± SD. The cut-off levels were determined as the mean + 3.8 × SD of the antibody activity in the exudates of healthy mice after Lardeux et al. [10].

### Statistical analysis

All data were calculated as mean ± standard deviation (SD). Statistical analyses were performed with GraphPad Prism version 5.00 and 7.00 (GraphPad Software, Inc., San Diego, CA, USA). Data were analyzed by either unpaired Student’s *t* test, one-way or two-way ANOVA followed by Dunnett’s or Bonferroni's multiple comparisons test to compare different groups. Differences were regarded as significant when *p* < 0.05.

## Results

### *Mesocestoides vogae* infection leads to the loss of the CD19b+ population from the peritoneal cavity of infected mice

**Fig. 1:**
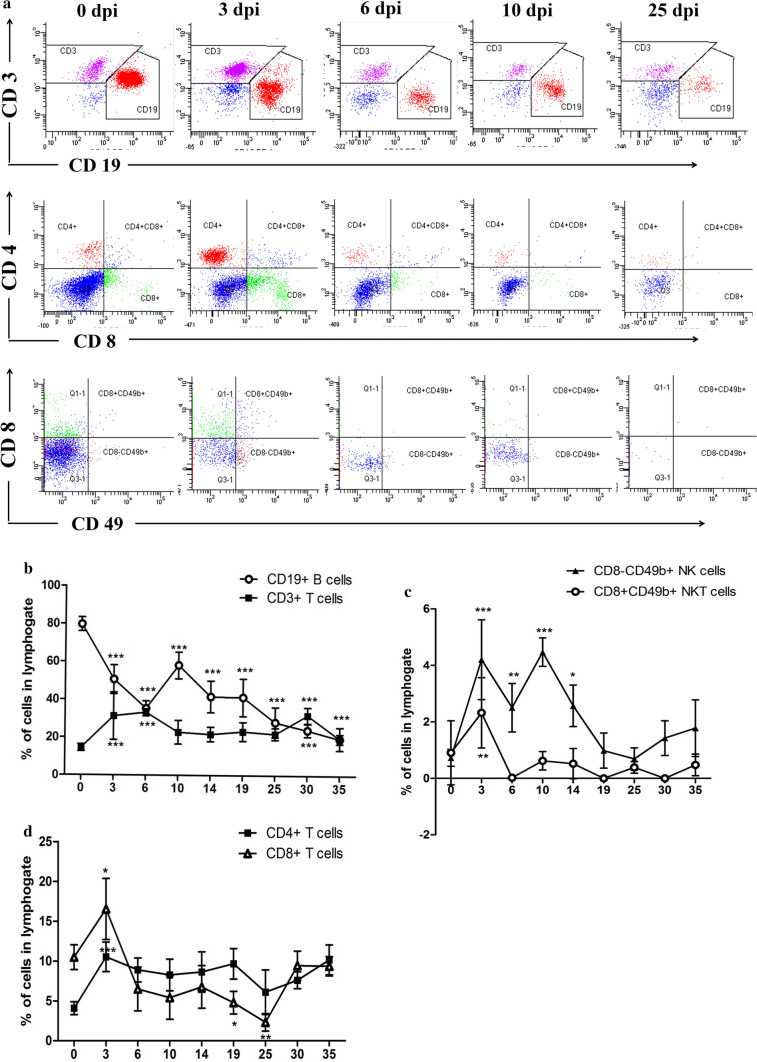
Peritoneal lymphocyte cell population in the peritoneal cavities of healthy and *Mesocestoides vogae*-infected mice at 3, 6, 10, 14, 19, 25, 30 and 35 days post-infection. Peritoneal exudate cells were isolated from the peritoneal cavity, and the expression of lymphocyte surface markers by these cells was analyzed from lymphogate using flow cytometry. **a** Representative dot plots show the expression of CD3/CD19, CD4/CD8 and CD49b/CD8 on PEC. Proportions of T- and B-lymphocytes (**b**), T-lymphocyte subpopulations (**c**), NK and NKT cells (**d**) in the peritoneal cavities of healthy and infected mice. Data are expressed as the means ± SD. Statistical significance was analyzed using one-way ANOVA with Dunnett's post-test, and significantly different values between control and infected groups are indicated as: *p < 0.05, **p < 0.01, ***p < 0.001.

To examine the kinetics of lymphocytes in the peritoneal cavity during *M. vogae* infection, we analyzed the expression of CD3, CD19, CD4, CD8 and CD49b markers within the population of peritoneal cells. The influence of *M. vogae* infection on the cellular composition/lymphocytes of the peritoneal cavity is shown in Fig. 1a. In general, upon experimental infection the percentage of peritoneal cells within the lymphogate significantly decreased from 57.96 ± 3.16% in healthy mice to 9.73 ± 1.49% at day 35 p.i. (*F*_(8,38)_ = 194.4, *p* < 0.0001; data not shown). The CD3+ T-cell percentage remained relatively stable, whereas the percentage of CD19+ B cells in the peritoneal cavity tended to decline, showing a four-fold decrease at day 35 p.i. [79.7 ± 3.67% *vs* 19.45 ± 2.52% control and 35 dpi, (*F*_(8,39)_ = 39.79, *p* < 0.0001; Fig. 1b). Furthermore, the CD8-CD49+ NK cells tended to be significantly higher in infected animals at days 3, 6 and 10 p.i. than in the healthy group (*F*_(8,28)_ = 14.5, *p* < 0.0001; Fig. 1c). Similarly, an increase in the percentage of CD8+CD49+ NKT cells (*F*_(8,35)_ = 7.267, *p* < 0.0001; Fig. 1c) and CD8+ T cells (*F*_(8,32)_ = 15.31, *p* < 0.0001; Fig. 1d) was seen at day 3 p.i. compared to the control group.

### *Mesocestoides vogae* infection is accompanied by a local production of IgG and IgM specific antibodies

**Fig. 2:**
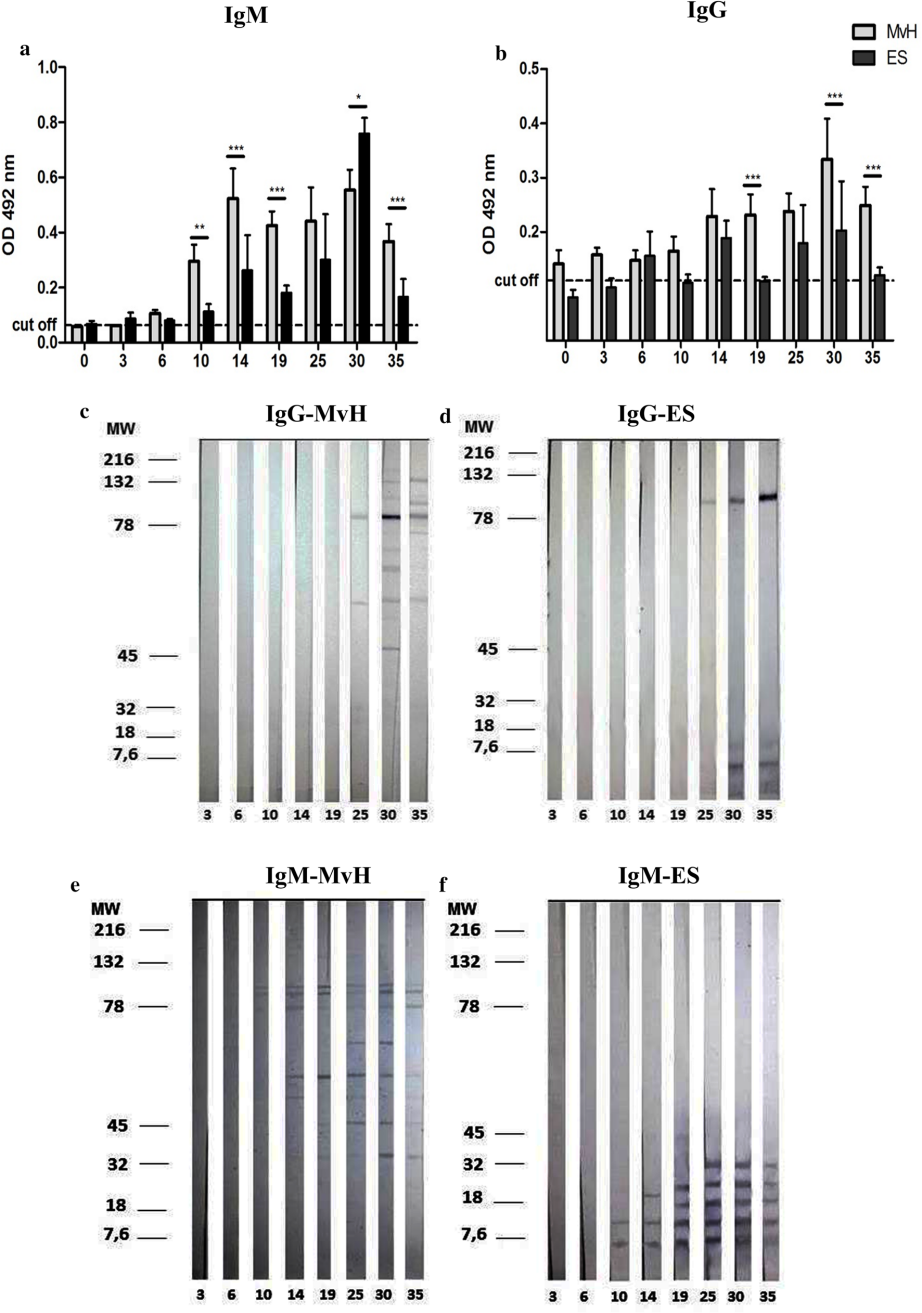
Antibody response of the IgM (**a**) and IgG (**b**) subclass specific to *Mesocestoides (M.) vogae* homogenate (MvH) or excretory/secretory molecules (ES) in the peritoneal exudates of healthy and infected mice at 3, 6, 10, 14, 19, 25, 30 and 35 days post-infection (p.i.). The anti-*M. vogae* IgM and IgG antibody titers were determined by ELISA methods. Data are presented as the means of OD ± SD. OD values of IgM for uninfected mice (*n* = 3) were 0.059 ± 0.004 for MvH antigen and 0.067 ± 0.012 for ES. OD values of IgG for uninfected mice (*n* = 4) were 0.141 ± 0.025 for MvH antigen and 0.079 ± 0.013 for ES. The cut-off values are indicated in the figures. Statistical significance was analyzed using two-way ANOVA followed by Bonferroni's multiple comparison tests, and significantly different values are indicated as: *p < 0.05, **p < 0.01, ***p < 0.001. Detection of immunogenic antigens within MvH and ES preparations immunoreactive to IgG (**c** MvH; **d** ES) and IgM antibodies (**e** MvH; **f** ES) performed by western blot analysis. Exudates from infected mice obtained on corresponding days p.i. were incubated with nitrocellulose strips with blotted MvH or ES antigens.

Antibody response in chronically infected mice is associated with hypergammaglobulinemia that is restricted to immunoglobulins M (IgM) and Gl (IgGl) [11, 12]. At the local level, very little is known about the kinetics of the host immune response and the role of local immunoglobulins. To investigate the immune profiles induced in mice by the *M. vogae* larvae, we measured the levels of IgG and IgM antibodies in the peritoneal fluid. As shown in Fig. 2a, the levels of IgM antibody to somatic larval *M. vogae* antigens (MvH) were significantly higher (0.29 ± 0.059 OD) than the level of IgM to ES (0.11 ± 0.02 OD) from day 10 p.i. However, the amount of IgM to ES antigens appears to be significantly increased at day 30 p.i. (0.75 ± 0.05 OD) compared to MvH (0.55 ± 0.07 OD, (*F*_(1,49)_ = 52.87, *p* < 0.0001). A higher level of specific anti-*M. vogae* IgG antibodies (Fig. 2b) to MvH was detected on day 14 p.i. (0.22 ± 0.05 OD) compared to the level of anti-MvH IgG detected in earlier days. Subsequently, the IgG titers to MvH slightly increased (0.23 ± 0.03 OD) and peaked on day 30 p.i. (0.33 ± 0.07 OD). The IgG titers to ES showed fluctuation in the peritoneal cavity of infected mice during the experiment. Interestingly, the mean absorbance of both antibody isotypes was significantly higher mainly for MvH than ES antigens probably because of the more immunogenic potential of MvH. Moreover, higher titers of peritoneal IgM against somatic antigen were detected from day 10 p.i., suggesting that this isotype may be predominant and involved in host protection, as was described in a model filarial infection [13].

In the peritoneal fluid from infected mice, the anti-MvH IgG antibodies to antigen with MW of approximately 89–90 kDa appeared on day 14 p.i. with increasing intensity up to day 30 p.i. (Fig. 2c). From this day bands corresponding to other immunoreactive parasitic proteins gradually appeared, showing different patterns on each day, and at least nine bands were detected on day 30 p.i. IgG antibodies reacted with three ES antigens forming one band with MW of approximately 90 kDa and two bands smaller than 7 kDa (Fig. 2d). The first anti-parasitic antibodies of the IgM isotype to MvH antigens were detected on day 10 p.i. forming two bands between 78–82 kDa (Fig. 2e). The number of bands with MW between 40 and 80 kDa increased with progressing infection, showing a different pattern on each day. A different profile of the peritoneal IgM antibody response to ES proteins (Fig. 2f) was observed from day 10 p.i., showing a ladder-like appearance, probably representing subunits of one larger dominant ES antigen.

### Peritoneal inflammation during *M. vogae* infection is associated with an increased number of myeloid CD11b+Gr-1+ cells

**Fig. 3:**
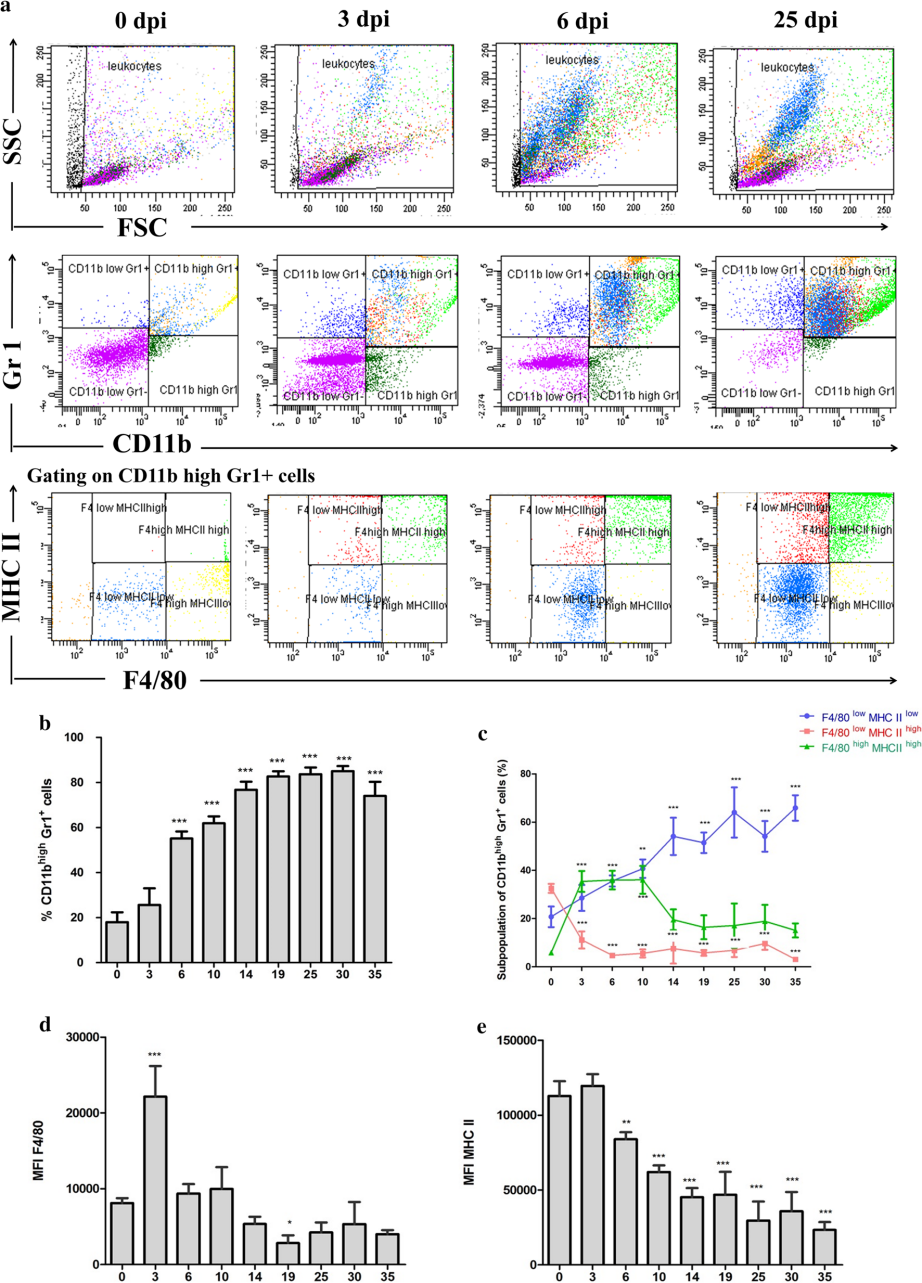
Peritoneal inflammation during *Mesocestoides (M.) vogae* infection is associated with an increased number of myeloid CD11b+Gr-1+ cells. Myeloid cell population in the peritoneal cavities of healthy and *M. vogae*-infected mice at 3, 6, 10, 14, 19, 25, 30 and 35 days post-infection. Peritoneal exudate cells (PEC) were isolated from the peritoneal cavity, and the expression of myeloid cell surface markers by these cells was analyzed using flow cytometry (FC). Live cells were gated on CD11b and Gr-1 expression to first identify CD11b+Gr-1+ cells. Then, F4/80 and MHC II expression was identified within the CD11b+Gr-1+ cells. **a** Representative dot plots obtained by FC analysis show the expression of CD11b/Gr-1 and F4/80/MHC II on PEC. **b** Proportions of CD11b^high^Gr-1+ cells in the peritoneal cavities of healthy and infected mice. **c** Proportions of F4/80+MHC II+ cells within CD11b^high^Gr-1+ cells in the peritoneal exudate cell population from healthy and infected mice. F4/80 (**d**) and MHC II (**e**) mean fluorescence intensity (MFI) within CD11b^high^Gr-1+ cells in the peritoneal exudate cell population from healthy and infected mice. Data are expressed as the mean ± SD. Statistical significance was analyzed using one-way ANOVA with Dunnett's post-test, and significantly different values between healthy and infected mice are indicated as: *p < 0.05, **p < 0.01, ***p < 0.001.

The peritoneal cavity is a dynamic compartment where many cell populations reside and interact [14]. Multiplication of metacestodes is accompanied by a significant recruitment of inflammatory cells into the site of infection [4]. The most prominent populations present in this compartment are myelo-monocytic cells of the macrophage phenotype, large granular cells, giant cells [4] and eosinophils [15]. To investigate the kinetics of cell accumulation in the peritoneal cavity, we performed multicolor flow cytometric analysis. We observed that the population of CD11b+Gr-1+ myeloid cells expanded in the peritoneal cavity progressively in the course of *M. vogae* infection, especially the percentage of cells co-expressing high levels of CD11b and Gr-1 (Fig. 3a, b). The percentage of CD11b^high^Gr1+ cells reached 17.97 ± 4.388% in healthy mice, while it significantly increased up to 85.02 ± 2.26% at day 30 p.i. (*F*_(8,38)_ = 151.1, *p* < 0.0001). It is obvious that not only the percentage but also the absolute number of CD11b+Gr-1+ cells was dramatically increased in mice with established infection. Similarly, we observed that the population of CD11b+Gr-1+ myeloid cells also expanded in the spleen of infected mice. However, the population of these cells expressed a lower level of CD11b (data not shown).

This myeloid cell population was further examined for surface expression of F4/80 and MHC class II molecules. We found that there were considerable changes in the expression levels of F4/80 and MHC II markers by myeloid cells (Fig. 3c). Compared with healthy mice (5.90 ± 0.98%), the percentage of CD11b+Gr-1+F4/80^high^MHC II^high^ cells (markers present on mature macrophages) significantly increased approximately seven-fold at day 3, 6 and 10 p.i. (36.08 ± 5.76%; *F*_(8,35)_ = 15.37, *p* < 0.0001). Later, the most prominent population was the CD11b^high^Gr-1+F4/80^low^MHC II^low^ cells, which significantly increased from 20.73 ± 4.27% in healthy mice to 64.00 ± 10.39 % at day 25 p.i. (*F*_(8,38)_ = 18.34, *p* < 0.0001). There was a significant upregulation of F4/80 expression and a mild increase in MHC II expression on CD 11b^high^ Gr1^+^ cells at day 3 p.i. compared to the control group (*F*_(8,37)_ = 39.46, *p* < 0.0001); however, the expression of both markers was subsequently downregulated (Fig. 3d, e), and most of this cell population expressed low levels of F4/80 and MHC II in later stages of infection.

### *Mesocestoides vogae* infection reduces inflammatory response in the peritoneal cavity and PEC continuously lost the characteristics of classically activated cells

**Fig. 4:**
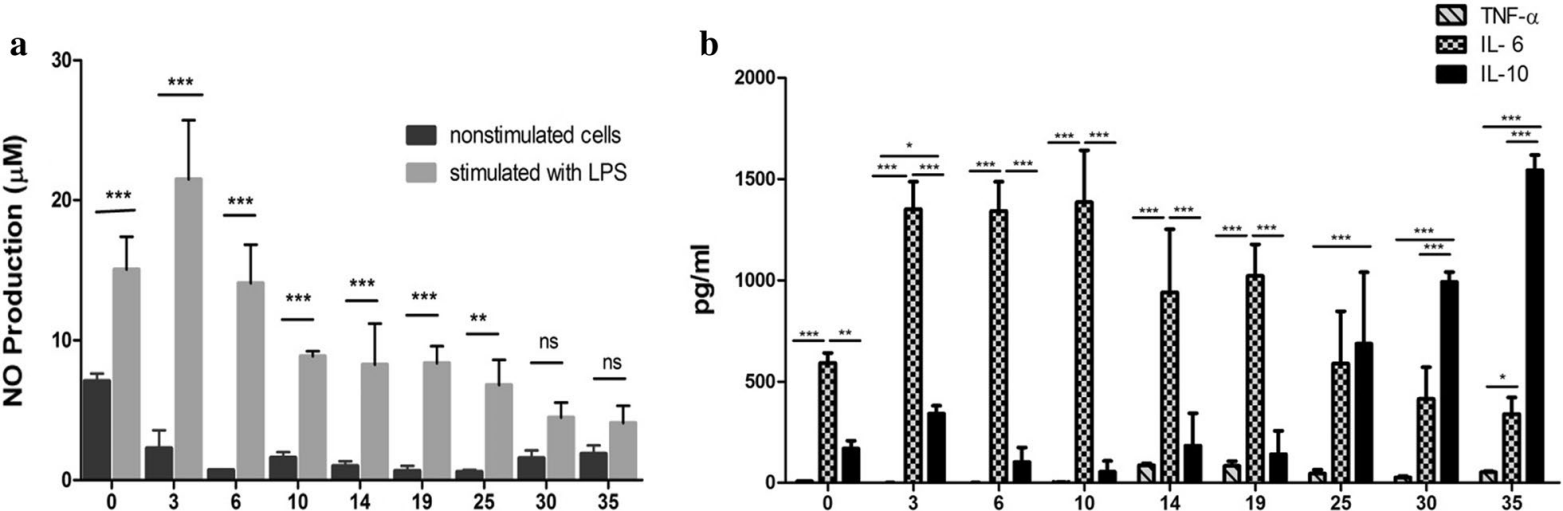
*Mesocestoides vogae* infection regulates the production of inflammatory cytokines in LPS-induced peritoneal exudate cells (PEC). PEC were isolated from the peritoneal cavity of healthy and infected mice at 3, 6, 10, 14, 19, 25, 30 and 35 days post-infection. Then PEC were cultured in 24-well plates in the presence or absence of LPS (1 µg/ml) at 37 °C 5% CO_2_ for 72 h. The concentration of NO (**a**) in the culture supernatants was determined as nitrite (NO_2_^−^) using Griess reagent. Nitrite concentration was determined using 0.1 M NaNO_3_ as the standard. Concentrations of cytokines TNF-α, IL-6 and IL-10 (**b**) in culture supernatants were measured by ELISA and expressed in pg/ml. Data are expressed as the mean ± SD. Statistical significance was analyzed using two-way ANOVA followed by Bonferroni's multiple comparison tests, and significantly different values are indicated as: *p < 0.05, **p < 0.01, ***p < 0.001.

The production of NO and inflammatory cytokines is associated with the stage of cell activation. Previously, Vendeľova et al. [4] demonstrated the ability of an *M. vogae*-derived ES product to induce directly the alternatively activated phenotype of PEC in vitro and in vivo, but the time kinetics of inflammatory responses from early to late stages has not been examined. To assess classical cell activation, the levels of cytokines IL-6, TNF-α, IL-10 and nitrite (NO) production were measured in culture supernatants of PEC ex vivo. The PEC were obtained in the times listed and exposed to 1 µg/ml of LPS for 72 h. Figure 4a shows that the NO level produced by LPS-stimulated PEC was significantly higher at day 0 and from 3 to 25 dpi compared to unstimulated PEC (*F*_(8,52)_ = 22.24, *p* < 0.0001). The PEC with or without stimulation isolated from mice at day 30 and 35 p.i. produced a lower level of NO. Significantly higher levels of inflammatory IL-6 (Fig. 4b) were produced by PEC isolated at the same points (from 3 to 25 dpi) as the level of TNF-α and IL-10 (*F*_(16,69)_ = 49.77, *p* < 0.0001). Moreover, exposure of PEC to LPS isolated later (at day 30 and 35. p.i) significantly stimulated the production of immunoregulatory IL-10. These findings suggest the presence of classically activated cells in the peritoneal cavity in the early stage of infection, likely found as CD11b+Gr-1+F4/80^high^MHC II^high^ cells. Although the effect of *M. vogae* products/antigens in the context of classical macrophage polarization was not assessed, it is likely that the increasing parasite burden with the accumulation of the ES antigens could directly regulate the production of proinflammatory and regulatory cytokines.

### *Mesocestoides vogae* infection expands myeloid cell populations that resemble MDSC

**Fig. 5:**
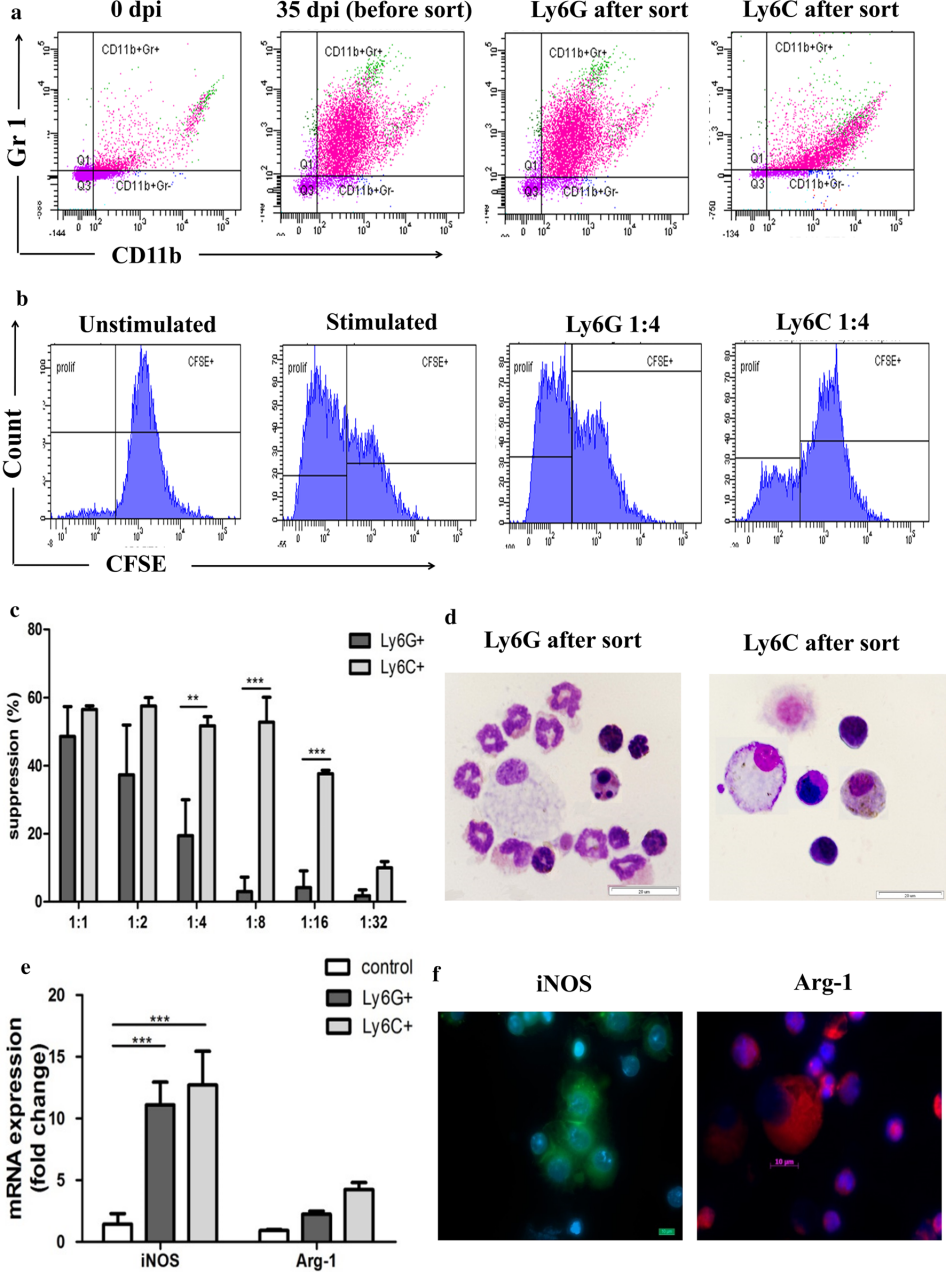
Peritoneal exudate cells (PEC) isolated from *Mesocestoides vogae*-infected mice possess suppressive capacities and can be subdivided into two subsets. Ly6C+ and Ly6G+ cells were isolated using MACS from the peritoneal cell population at day 35 post-infection and cultured with CFSE-labeled T cells stimulated with anti-CD3/ anti-CD28 mAbs for 72 h at different ratios. **a** Flow cytometry plots of PEC from healthy and infected mice showing the expression of CD11b and Gr-1 before and after MACS sort. **b** Representative histograms showing suppression of spleen T cells by sorted Ly6C+ or Ly6G+ in vitro (1:4 ratio MDSC to T cells). **c** The graph represents the proliferation inhibition of CD3/CD28-activated spleen T cells (the ratio of myeloid cell subsets to T cells as indicated in the graph). Results represent means ± SD of triplicates. Statistical significance was analyzed using Student's *t* test, and significantly different values are indicated as: **p < 0.01, ***p < 0.001. **d** The sorted cells were subjected to May-Grünwald/Giemsa staining. Representative pictures showing the morphology of sorted subsets are depicted. Original magnification, ×1000, scale bar = 20 μm. **e** mRNA expression of iNOS and Arg-1 in PEC of healthy mice (control) and sorted Ly6C+ or Ly6G+ cells. Statistical significance was analyzed using two-way ANOVA followed by Bonferroni's multiple comparison tests and significantly different values are indicated as: ***p < 0.001. **f** Immunofluorescent assay of iNOS and Arg-1 in whole peritoneal cells. Hoechst staining was used to visualize nuclei. Original magnification, ×1000, scale bar = 10 μm.

To further characterize *M. vogae*-induced CD11b+Gr-1+ cells and to determine their similarities to MDSC subpopulations, an in vitro T-cell proliferation assay was performed. It is known that MDSC are a very heterogeneous cell population that can be generally divided into a monocytic (Ly6C+) and granulocytic (Ly6G+) subset. In this work, we focused on the suppression of CD3+ T cells, as they are basically used to evaluate the function of suppressor cells, and it is the gold standard for identifying MDSC. Ly6C+ and Ly6G+ cells were isolated using MACS from the peritoneal cell population at day 35 p.i. (Fig. 5a). To measure the suppressive capacity, both MACS-isolated subsets were cultured individually with pre-activated T cells for 72 h at different ratios. As shown in Fig. 5b, c, both subsets markedly suppressed CD3+ T-cell proliferation in response to anti-CD3/CD28 stimulation in a 1:1 MDSC to T-cell ratio. The strongest inhibition was detected using a Ly6C+:T-cell ratio of 1:2, where > 57% suppression was observed. Ly6C+ subsets also remained significantly suppressive at a higher 1:8 MDSC:T-cell ratio, suggesting Ly6C+ cells have a stronger suppressive activity compared to Ly6G+ cells (*F*_(5,15)_ = 7.63, *p* = 0.0034). Moreover, there was a significant inhibition in the proliferative response (37.62 ± 1.05% suppression relative to control) even when the Ly6C:T-cell ratio was 1:16. Analysis of stained cell smears (Fig. 5d) confirmed that the majority of Ly6G+ cells recovered had either segmented nuclei or constricted ring-shaped nuclei, which are seen in polymorphonuclear cells and their precursors. Most cells had granular cytoplasm and a few had eosinophilic granules (eosinophils). Cells in the Ly6C+ population had an immature myelocyte/mononuclear appearance with more abundant basophilic cytoplasm. Monocytic and granulocytic subpopulations of myeloid cells have different mechanisms of their suppression [16]. Therefore, we next examined the level of iNOS and Arg-1 in both subsets (Fig. 5e). iNOS and Arg-1 were expressed within both subsets, but at a lower level in the Ly6G+ cells than the Ly6C+ cells (11.09 ± 1.84 *vs* 12.71 ± 2.72 for iNOS, 2.24 ± 0.24 *vs* 4.24 ± 0.56 for Arg-1; *F*_(2,3)_ = 15.94, *p* < 0.001). To verify the presence of macrophages and other myeloid cells in the peritoneum of *M. vogae*-infected mice, we performed iNOS and Arg-1 immunofluorescence staining at day 35 p.i. (Fig. 5f). iNOS was abundant in macrophages/mononuclear cells and appeared to be scattered throughout the cytoplasm of these peritoneal cells, whereas the most intense Arg-1 signal occurred as granules in polymorphonuclear cells with segmented nuclei and at much lower levels in macrophages.

### IL-10 production with STAT-3 mRNA expression is upregulated after infection

The larval stages of *M. vogae* can reproduce asexually in the liver and serosal cavities of their intermediate hosts, mainly in the peritoneal cavity [17, 18]. The numbers of larval stages in the peritoneal cavity significantly increased (*p* < 0.01) at day 25 p.i. (487.7 ± 96.13) compared to day 3 p.i. (7.600 ± 5.320) (*F*_(7,38)_ = 98.77, *p* < 0.0001; data not shown).

**Fig. 6:**
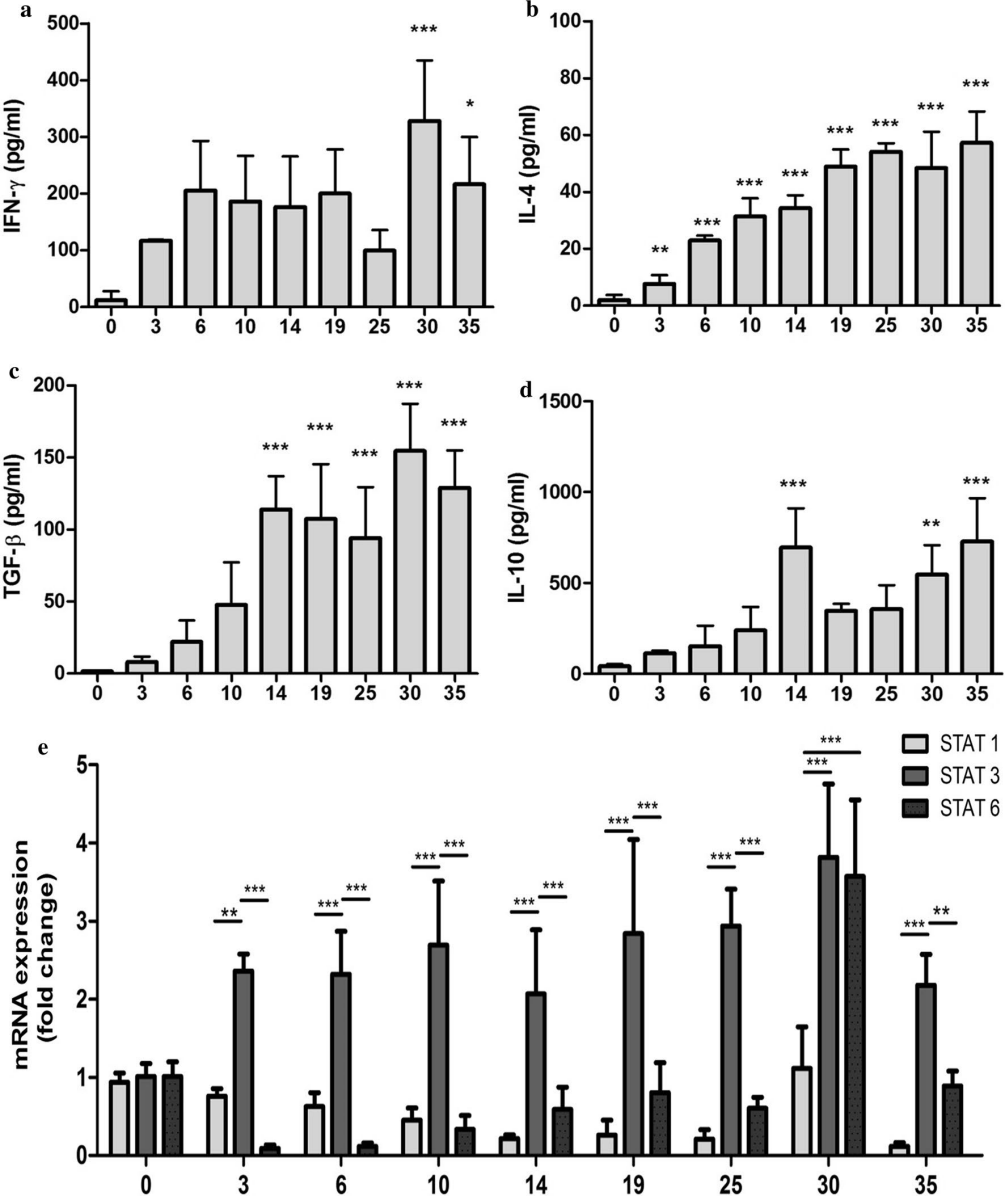
*Mesocestoides vogae* infection is associated with IL-10 production. Concentrations of cytokines IFN-γ (**a**), IL-4 (**b**), TGF-β (**c**) and IL-10 (**d**) in the peritoneal exudates of healthy and *Mesocestoides vogae*-infected mice at day 3, 6, 10, 14, 19, 25, 30 and 35 post-infection were measured by ELISA and expressed in pg/ml. Data are expressed as the mean ± SD. Statistical significance was analyzed using one-way ANOVA with Dunnett's post-test and significantly different values between healthy and infected mice are indicated as: *p < 0.05, **p < 0.01, ***p < 0.001. **e** Relative expression of m-RNA for STAT-1, STAT-3 and STAT-6 in the peritoneal exudate cells of healthy and infected mice was analyzed by real-time PCR. Data are expressed as means ± SD after comparison with uninfected PEC following normalization to β-actin. Statistical significance was analyzed using two-way ANOVA followed by Bonferroni's multiple comparison tests, and significantly different values are indicated as: **p < 0.01, ***p < 0.001.

To understand how *M. vogae* infection in mice is linked with the local release of pro- and anti-inflammatory cytokines, we analyzed the cytokine response in the peritoneal compartment. The level of IFN-γ, IL-4, TGF-β and IL-10 in the peritoneal lavage fluid from healthy and infected mice was detected by ELISA. IFN-γ (Fig. 6a) levels began to increase at day 3 p.i. (117.3 ± 1.476 pg/ml) and reached a significant peak at day 30 p.i. (328.5 ± 106.5 pg/ml; *F*_(8,25)_ = 4.56, *p* = 0.0016). Compared to day 0, significant changes in the concentration of IL-4 (Fig. 6b) were detected at day 3 p.i. (7.695 ± 3.024 pg/ml; *F*_(8,33)_ = 32.04, *p* < 0.0001) and then gradually increased with the progression of the infection. A similar trend was observed for the level of TGF-β (Fig. 6c), with significant elevation from day 14 p.i. (114.0 ± 23.10 pg/ml; *F*_(8,34)_ = 24.73, *p* < 0.0001). One of the most significantly upregulated cytokines within PEC and spleen cells following *M. vogae* infection is anti-inflammatory IL-10 [4]. Our study confirmed that metacestode infection elicits high levels of IL-10 production in infected mice (Fig. 6d). IL-10 production began to increase in the peritoneal cavity on day 3 p.i., and significant changes in the level of IL-10 were seen at day 14 p.i. (695.8 ± 215.6 pg/ml; *F*_(8,29)_ = 10.85, *p* < 0.0001). Of this cytokine profile, IL-10 became the dominant cytokine in the peritoneum and appears to be a major factor in regulating immunity.

Recent work has described a critical role for STAT-6 in the upregulation of the alternative activation markers, which is required for controlling *M. corti*-induced neurocysticercosis [19]. To further assess the role of STAT within the milieu of peritoneal cells and to examine their regulation of expression during experimental infection, PEC were isolated and molecular analysis was performed. Compared to the control, expression levels of STAT-1 and STAT-6 were initially downregulated early during the infection at day 3 p.i., whereas STAT-3 expression was significantly elevated and remained at high levels until the end of the observation period (*F*_(16,68)_ = 5.60, *p* < 0.0001; Fig 6e). STAT-1 mRNA expression continuously declined during infection from 0.93 ± 0.11-fold in the healthy control to 0.11 ± 0.05-fold at day 35 p.i., except for day 30 p.i. (1.11 ± 0.53-fold), probably as a response to a significantly higher level of IFN-γ in the peritoneal cavity. STAT-6 mRNA, which was constitutively expressed at low levels in infected mice, was upregulated on day 25 p.i. (1.41 ± 1.41-fold), increasing to maximal levels by day 30 p.i. (3.57 ± 0.97-fold). Only the mRNA level of STAT-3 was consistently higher and reached the peak at day 30 p.i. (3.81 ± 0.93-fold), suggesting a critical role in anti-inflammatory reactions mediated by IL-10.

### Excretory-secretory products of *M. vogae* recruit CD11b+IL-10+ PEC

**Fig. 7:**
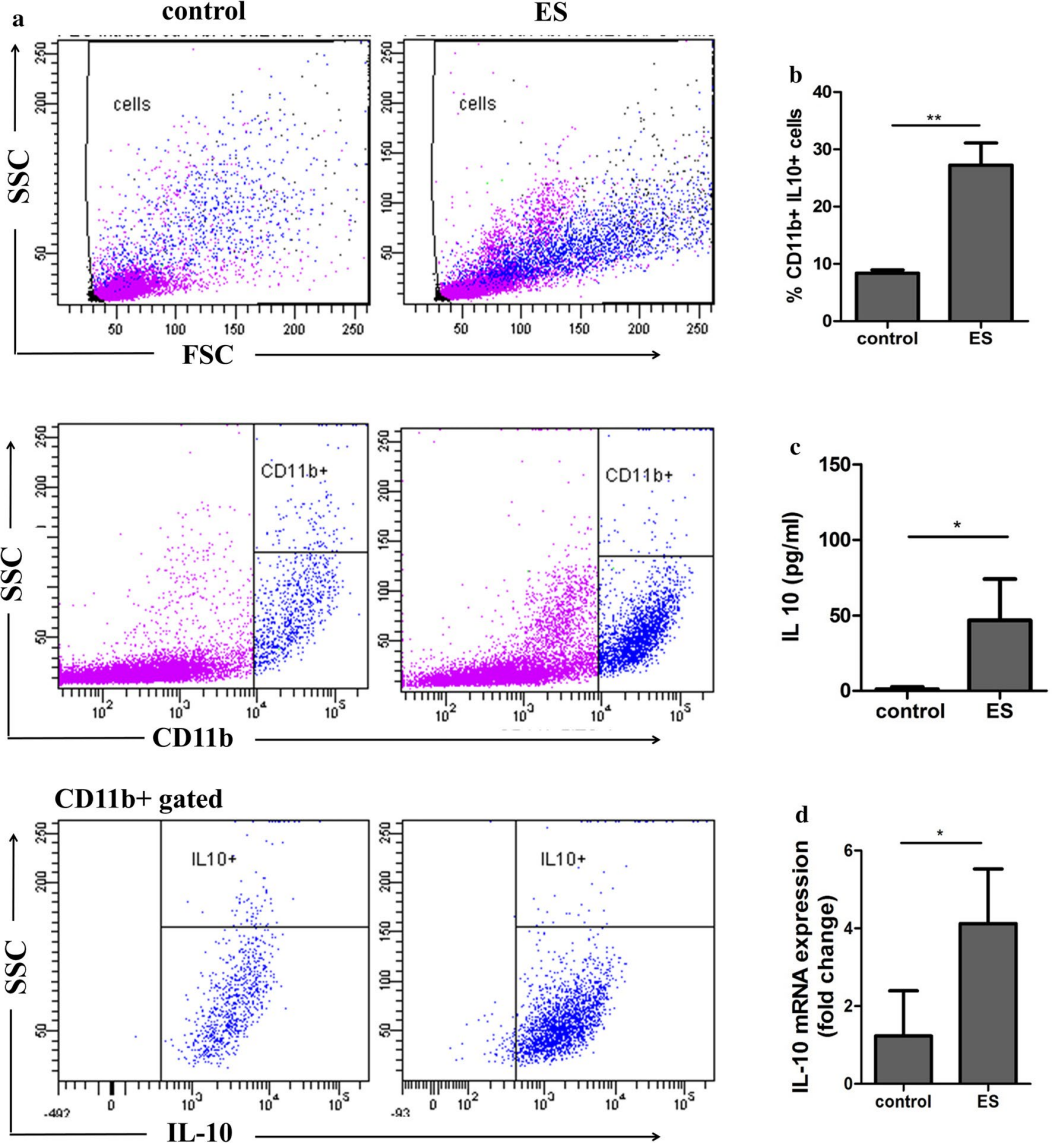
*Mesocestoides vogae* excretory-secretory (ES) products induce the accumulation of CD11b+IL-10+ peritoneal cells. Mice were injected intraperitoneally with 20 µg of ES and PBS as control every day for a total of 6 days. Peritoneal exudate cells (PEC) were isolated and subjected to flow cytometric analysis. **a** Representative flow cytometry plots showing the expression of IL-10 and CD11b by PEC. Live cells were gated on CD11b expression to first identify CD11b+ cells. Then, IL-10 expression was identified within CD11b+ cells. **b** Proportions of CD11b+IL-10+ cells in the peritoneal cavities of PBS and ES treated mice. **c** Concentrations of IL-10 in the peritoneal exudates was measured by ELISA and expressed in pg/ml. **d** Relative expression of m-RNA for IL-10 in the peritoneal exudate cells was analyzed by real-time PCR. Data are expressed as the mean ± SD. Statistical significance was analyzed using Student's *t* test, and significantly different values between healthy and ES treated mice are indicated as: *p < 0.05, **p < 0.01.

Previous studies have established that helminth infection or its molecules are associated with the increased presence of a myeloid CD11b+Gr1+ cell population that has a suppressive capacity [20–24]. Next, we examined the peritoneal cell influx and fluid following the injection of parasite ES antigens. To examine these effects, Balb/c mice were injected intraperitoneally with 20 µg of ES preparation in six doses; PEC were isolated and CD11b+cells were assessed for IL-10 localization (Fig. 7a, b). Intracellular cytokine staining showed low proportions of IL-10-expressing CD11b+ cells in the peritoneal cavity of healthy mice (8.4 ± 0.56%). After injection of the ES preparation, a significantly higher proportion of CD11b+IL-10+ was detected (27.25 ± 3.851) compared to the control (*F*_(3,1)_ = 46.34, *p* = 0.0029), and the majority of cells expressing a higher level of CD11b (89.8%) were IL-10+ (data not shown). Moreover, the level of IL-10 tested by ELISA was significantly higher in the peritoneal fluid of mice injected with ES preparations (1.13 ± 1.54 *vs* 46.84 ± 27.04 pg/ml, control *vs* ES, *F*_(1,2)_ = 306.1, *p* = 0.0496; Fig. 7c). IL-10 mRNA expression (Fig. 7d) was also significantly elevated within PEC after the ES injections (1.23 ± 1.15 *vs* 4.12 ± 1.40, control *vs* ES, *F*_(4,3)_ = 1.427, *p* = 0.0131). The expression of IL-10 by CD11b+ cells suggests that myeloid populations are the major source of these immunoregulatory mediators and that ES products seem to be the major factor during *M. vogae* infection, not excluding the role of some MvH.

## Discussion

In our study, an experimental model for larval cestodiasis is presented. Using an *M. vogae* model we were able to study the effect of metacestodes on hosts immune status and cellular composition of the peritoneal cavity. Under steady-state conditions, the resident peritoneal cell population includes macrophages, neutrophils and NK cells as well as T and B lymphocytes. NK cells are important in immune defense against infections caused by viruses, bacteria and protozoa. However, their role in helminth infections is not well documented. Our results showed that there is an increase in the percentage of NK cells (3–10 day p.i.), NKT cells and cytotoxic CD8+ T cells (at day 3 p.i.) in the peritoneal cavity of mice in the early phase of *M. vogae* infection. Similarly, in the experimental model of *E. granulosus* infection, kinetic analyses showed a rapid increase in peritoneal NK cells and cytotoxic T cells peaking at day 5 and 7 p.i. [25]. This finding suggests that cell-mediated cytotoxicity may play important roles in helminth infections, although parasite-induced proteins might interfere with the effector functions of these cells and thereby contribute to the downregulation of the host immune response [26]. *M. vogae* infection was also marked by a decline of peritoneal CD19+ cells possibly due to their differentiation into plasma cells, as evidenced by increased local IgM and IgG secretion. B cells and their antibody products play a crucial role in the antiparasitic immunity of serosal cavities, where they proliferate and produce antigen-specific IgM [27]. In the experimental *E. granulosus* infection, B lymphocytes were shown to undergo an early plasma cell differentiation process, which was confirmed by altered regulation of key factors (Pax5, Bcl-6, Blimp-1) and functionally by the local secretion of antigen-specific IgM and IgG2b antibodies [25]. Metacestode surface-bound antibodies can be involved in a parasite evasion strategy. In our study, the higher levels of IgM antibodies in comparison with IgG to both MvH and ES antigens were detected in the peritoneal fluid, and marked IgM elevation was observed from day 10 p.i., which corresponded to the appearance of several immunoreactive bands using the western blot technique. A different profile of specific IgM-immunoreactive bands was found for ES antigens, suggesting that IgM antibodies to parasitic antigens may participate in developing the suppressive environment in the peritoneum. Our results confirm the stage-specific protein expression by western blot analysis in *M. vogae* metacestode infection.

Another cell population that increased in response to *M. vogae* infection is CD11b+Gr-1+ cells. Similarly, this phenomenon was also induced in mice infected with *Taenia crassiceps* [24] and *Litomosoides sigmodontis* filarial infective larvae [28, 29]. Moreover, myeloid populations can be mobilized by parasitic helminth-derived antigens [30, 31]. In our study, these cells were also found to possess additional cell surface markers like F4/80 and MHC II expressed at a low level, indicative of a recruited monocyte-derived population [28] and/or inflammatory monocytes [32]. On the other hand, Th2-biased response is characterized by proliferation of tissue macrophages rather than recruitment from bone marrow [29]. Moreover, we found that *M. vogae* infection influenced the expression of F4/80 and MHC II markers on the myeloid peritoneal cell population. Recently, we showed that peritoneal myeloid cells undergo apoptosis in response to *M. vogae* infection [33] and that ES products can directly inhibit the activation of antigen presenting cells [34], which may mediate immunosuppression. The alternation of MHC II and F4/80 expression levels in PEC demonstrates the complex network of interactions and regulatory mechanisms that occur during an experimental infection.

Because the helminth parasites and their ES product biased immune response to the anti-inflammatory type, the therapeutic potential has been considered in many inflammatory disorders and autoimmune diseases [35, 36]. In our study, peritoneal macrophage cell lines were assessed for TNF-α, IL-6 and IL-10 secretion following LPS stimulation. We observed that metacestode infection decreased the secretion of proinflammatory cytokines and induced the production of IL-10 by peritoneal cells. LPS-stimulated PEC isolated at a later stage of infection were not able to produce inflammatory cytokines IL-6 and TNF-α. As the infection progressed, PEC/macrophages from infected mice also showed an impairment of their ability to secrete NO in response to LPS compared to PEC obtained during the early phase of infection (first week). Although *M. vogae*-derived products induce the expression of markers of alternative activation (YM1, Arg-1 and Fizz-1) in vivo and in vitro [4], it is likely that the parasite ES antigens play important role in regulating cytokine production by inhibiting their transcripts, as has been previously reported [37].

Recent studies have described that the heterogeneous population of immature myeloid cells plays a crucial role in the regulation of adaptive immunity. There has been considerable interest in a population of cells recently termed myeloid-derived suppressor cells (MDSC), which have been shown to express CD11b and Gr-1 (Ly6G/Ly6C) markers in mice and inhibit T- and/or B-cell proliferation [38, 39]. MDSC in mice comprise two subsets corresponding to monocytic and granulocytic (polymorphonuclear) cells, which differ in Ly6C and Ly6G expression profiles. Moreover, MDSC have a low or undetectable expression of mature antigen-presenting cell markers, such as MHC II molecules and F4/80. Both subsets induced suppression of immune responses through several pathways, mainly L-arginine depletion through Arg-1 and iNOS activity, increased generation of reactive oxygen and nitrogen species and production of immunosuppressive and immunoregulatory cytokines, such as TGF-β and IL-10 [40]. Thus, we also hypothesized that CD11b+Gr-1+ cells induced by *M. vogae* tetrathyridia may contribute to the suppression of adaptive immune response at the systemic level, as described in other infection parasitic models [21, 24]. Using standard in vitro immune suppression assays, both peritoneal Ly6C+ and Ly6G+ subsets significantly inhibited CD3+ T-cell proliferation in response to anti-CD3/CD28 stimulation, with the suppression by Ly6C+ cells being more efficient. Expressions of Arg-1 and iNOS by MDSC have been shown to be associated with their immunosuppressive function. Analysis of Arg-1 and iNOS expression level demonstrated that subpopulation of Ly6C+ cells expressed both markers at higher level, suggesting their higher immunosuppressive potential. Most relevant to our findings, a recent study by Brys et al. [24] demonstrated in a *Taenia crassiceps* model that adherent peritoneal cells mediated their suppression through their secretion of NO (in the early stage) and production of ROS via arginase activity (in the late stage of infection). Arg-1 and iNOS co-expression has also been described in monocytic MDSC, which is present in the heart during the acute phase of *Trypanosoma cruzi* infection [41]. These data demonstrate that CD11b+Gr-1+ cells from the peritoneal cavity can suppress T-cell proliferation *in vitro*, confirming that they can be important regulators of immunity. Regarding this suggestion, further investigations are needed to better understand the function of myeloid cells in *M. vogae* infection.

The immune response against this parasite is driven by excretory-secretory molecules produced by tetrathyridia lying in the host’s peritoneal cavity and liver. It has been reported that IL-4 plays an important role in being the key mediator in the host immune response elicited by *M. vogae* larvae [8, 42]. Recently, Vendelova et al. [4] showed that there is an early and systemic Th2-type cytokine response with increasing IL-10 mRNA level, which is probably unrelated to protective immunity. Similarly, in our study we found that anti-inflammatory IL-10 produced by peritoneal leukocytes is the main component in the cytokine milieu surrounding these parasites in the late stage of infection. In addition, peritoneal IL-4 and TGF-β levels were significantly increased in the presence of CD11b^high^Gr-1+ myeloid cells expressing a low level of activation and maturation markers at day 14 p.i.

The binding of a wide range of growth factors and cytokines to their receptors located on the cell surface activates the receptor-associated Janus kinases (JAK) and subsequently leads to the phosphorylation of the cytokine receptor complex. Thereafter, the binding and activation of signal transducer and activator of transcription (STAT) proteins induce their translocation to the cell nucleus for signal transduction and the initiation of the gene transcription [43]. While the IFN/STAT-1/STAT-2 signaling pathway is mostly associated with immune activation, other STAT proteins, like STAT-3 and STAT-6, take part in the suppression of immune responses and inflammation by mediating the effects of IL-10 and IL-4 [44]. Analysis of the mixed population of PEC by qPCR detected increased transcription of STAT-3 and in the later stage of infection also STAT-6, which are associated with anti-inflammatory processes. The STAT-6 dependent pathway is particularly important in central nervous system (CNS) immunity during *M. corti* infection and in the development of alternative activated cells, where a deficiency in STAT-6 led to increased parasite load and decreased survival [19]. STAT-3 is the primary mediator in the IL-10 signaling pathway, an immunoregulatory cytokine that plays a fundamental role in maintaining the activation/deactivation balance of mononuclear cells through the inhibition of expression of LPS-inducible genes and expression of antigen-presenting surface markers [45]. Moreover, IL-10 can directly inhibit IFN-γ-induced gene expression in monocytes by downregulating STAT-1 activation [46]. This cytokine can be produced by almost all cell populations of both innate (monocytes, macrophages, neutrophils, dendritic cells, NK cells) and/or adaptive immunity (T and B cells) [47, 48]. In our study, injections of parasite ES molecules directly induced the production of IL-10 and the development of CD11b+IL10+ cells. These results are in agreement with previous studies showing that IL-10 produced by innate cells or T reg is crucial to downregulate the immune response to other helminths, such as schistosomes [49] or the intestinal helminth *Heligomosomoides polygyrus* [50]. It is possible that parasite-primed CD11b+ myeloid cells may contribute to a dysfunctional peritoneal immunity by creating a cytokine milieu that fails to develop a protective Th1 response.

## Conclusions

In summary, *M. vogae* metacestodes induced the accumulation of immature myeloid CD11b+Gr1+ cells in the peritoneal cavity of infected mice that resemble MDSC. The increasing parasite burden was associated with a higher IL-10 level and consistently upregulated the expression of STAT-3, which could be one of the regulatory mechanisms in *M. vogae* infection to control the release of pro-inflammatory cytokines. Understanding the molecular interactions between the parasite and its host that drives immune modulation could lead to the development of new strategies for managing metacestode infections.

## Data Availability

The datasets used and analyzed during the present study are available from the corresponding author upon reasonable request.

## Abbreviations

Arg-1: Arginase 1
CM: Complete medium
ES: Excretory-secretory products
IFN-γ: Interferon γ
Ig: Immunoglobulins
IL: Interleukin
iNOS: Inducible nitric oxide synthase
LPS: Lipopolysaccharide
MACS: Magnetic-activated cell sorting
MDSC: Myeloid-derived suppressor cells
MHC II: Major histocompatibility complex
MvH: *Mesocestoides vogae* Homogenate
NK cells: Natural killer cells
NO: Nitric oxide
p.i.: Post-infection
PEC: Peritoneal exudate cells
RPMI: Roswell Park Memorial Institute
STAT: Signal transducer and activator of transcription
TGF-β: Transforming growth factor β
TNF-α: Tumor necrosis factor α
